# Gender differences in prolactin thresholds and their association with lactotroph adenoma invasiveness for potential treatment considerations

**DOI:** 10.1038/s41598-025-90250-6

**Published:** 2025-03-20

**Authors:** Lukas Andereggen, Emanuel Christ, Andrea Stieger, Markus M. Luedi, Markus Huber

**Affiliations:** 1 Department of Neurosurgery, Kantonsspital Aarau, Aarau, Switzerland; 2 Faculty of Medicine, University of Bern, Bern, Switzerland; 3 Department of Endocrinology, Diabetes and Metabolism, University Hospital of Basel, Basel, Switzerland; 4 Department of Anaesthesiology and Pain Medicine, Kantonsspital St. Gallen, St. Gallen, Switzerland; 5 Department of Anaesthesiology and Pain Medicine, Bern University Hospital, Inselspital, University of Bern, Bern, Switzerland

**Keywords:** Dopamine agonists, Long-term outcome, Machine learning, First-line surgery, Prolactinoma, Prediction modeling, Predictive markers, Endocrine system and metabolic diseases, Outcomes research

## Abstract

Recent trends in first-line transsphenoidal surgery (TSS) for prolactinoma patients aim to reduce long-term dependence on dopamine agonists (DA). Key factors linked to poor surgical outcomes include cavernous sinus invasiveness and high baseline serum prolactin (PRL) levels. Defining simple PRL threshold values to indicate invasiveness and inform treatment strategy is crucial. In this retrospective cohort study of 149 prolactinoma patients treated with first-line transsphenoidal surgery (TSS) or dopamine agonist (DA) therapy, we evaluated preoperative prolactin (PRL) levels and cavernous sinus invasion as factors associated with long-term remission. Bayesian modeling identified cohort-wide and gender-specific PRL thresholds associated with invasiveness. Preoperative PRL values strongly correlated with cavernous sinus invasion (AUROC = 0.95; 95% CI: 0.90–0.98). The cohort-wide PRL threshold was 431.9 µg/L (95% CI: 181.1–708.3 µg/L), with gender-specific thresholds of 280.8 µg/L (95% CI: 51.0–528.2 µg/L) for women and 1325.0 µg/L (95% CI: 667.2–2582.9 µg/L) for men. Female thresholds were lower and less affected by age and obesity, while male thresholds were influenced by these factors, particularly in young, obese men. These findings suggest that gender-specific PRL thresholds may be useful for improving specificity and sensitivity in identifying invasiveness, potentially aiding clinical decisions. Personalized treatment informed by preoperative biomarkers is essential for optimizing outcomes and reducing DA reliance, but it should be considered in conjunction with a comprehensive clinical evaluation.

**Supplementary Information:**

The online version contains supplementary material available at 10.1038/s41598-025-90250-6.

Email: markus.huber@ insel.ch.

## Introduction

Prolactinomas pose unique therapeutic challenges for clinicians, as predicting sustained long-term remission rates for both upfront surgery and medical therapy remains difficult^1,2^. As of today, personalized therapy tailored to individual prolactinoma patients, involving interdisciplinary consensus and personal patient discussion, can help guide the ultimate decision towards optimal treatment. This is especially crucial given the absence of randomized trials concerning the primary first-line treatment strategy and its long-term outcomes^3^. While both dopamine agonists (DAs) and first-line transsphenoidal surgery (TSS) are effective treatment options, concerns about adverse effects and long-term reliance (i.e., dependence) on DAs are not uncommon^4–13^. As a result, first-line surgery is increasingly offered to younger patients with microprolactinomas or macroprolactinomas not invading the cavernous sinus^14–16^, in line with the Pituitary Society’s 2024 consensus statement^17^. Thereby, both elevated baseline prolactin (PRL) levels and cavernous sinus invasion are consistent determinants of prolactinoma diagnosis and treatment outcomes^14,18^. Therefore, in clinical practice, simple PRL threshold values are warranted to help define invasiveness and inform treatment decision between TSS and DA therapy in the aim of achieving long-term cure. Considering the gender differences in tumor size, particularly with microadenomas being more prevalent in women^19^, and the possibility that higher BMI levels might be linked to prolonged exposure to hyperprolactinemia^20^, our objective was to establish gender-specific PRL thresholds for assessing adenoma invasiveness. This aimed to reduce long-term reliance on DAs and to provide additional insight to guide clinical decision-making process toward the optimal treatment option in prolactinoma patients.

## Results

### Cohort description

A total of 56 (37.6%) out of 149 patients exhibited cavernous sinus invasion. Among them, 62 (41.6%) patients underwent medical treatment, while 87 (58.4%) patients opted for upfront surgery. Adenoma size was evenly distributed across the entire cohort, with only macroadenomas diagnosed in patients with cavernous sinus invasion. The majority of patients were female (71.1%), with a median age of 33 years (interquartile range (IQR): 27–47 years). The median BMI was 26.8 kg/m^2^ (IQR: 21.9–30.1), with patients displaying cavernous sinus invasion having higher BMI values (median of 27.7 kg/m^2^ versus 24.9 kg/m^2^, unadjusted p-value: 0.001). In the long term, 74 patients achieved remission without the need for persistent dopamine agonist (DA) therapy following upfront surgery, and 10 patients following primary DA therapy (unadjusted p-value: 0.068). At the last follow-up, 80 patients were still on DA therapy (primary surgery vs. primary medical therapy, unadjusted p-value: <0.001) (Table 1).

**Table 1:** Cohort description.

	Primary outcome: Cavernous sinus invasion				
	All patients	No invasion	Invasion	*p*	N
	*N* = 149 *(62.4%)*	*N = 93/149* *(62.4%)*	*N = 56/149* *(37.6%)*		
Primary treatment, TSS (n/%)	87 (58.4%)	65 (69.9%)	22 (39.3%)	< 0.001	149
Adenoma size, Macro (n/%)	76 (51.0%)	20 (21.5%)	56 (100%)	< 0.001	149
Sex, female (n/%)	106 (71.1%)	85 (91.4%)	21 (37.5%)	< 0.001	149
Age (years) (Median, IQR)	33.0 [27.0–47.0]	31.0 [27.0–41.0]	44.5 [27.0–55.5]	0.001	149
Headache (n/%)	42 (29.4%)	17 (19.1%)	25 (46.3%)	0.001	143
Gonadotropin deficiency (n/%)	32 (28.6%)	5 (8.06%)	27 (54.0%)	< 0.001	112
Partial hypopituitarism (n/%)	36 (26.5%)	9 (10.7%)	27 (49.1%)	< 0.001	136
BMI (kg/m^2^) (Median, IQR)	26.8 [21.9–30.1]	24.9 [21.2–27.6]	27.7 [25.2–31.5]	0.001	102
PRL levels (µg/L) (Median, IQR)	216 [102–1072]	126 [79–202]	1,510 [745–4,561]	< 0.001	131
DA therapy (n/%)	62 (41.6%)	28 (30.1%)	34 (60.7%)	< 0.001	149
Bromocriptine (Parlodel^®^)	31 (20.8%)	16 (17.2%)	15 (26.8%)	0.235	149
Cabergoline (Cabaser^®^)	18 (12.1%)	5 (5.38%)	13 (23.2%)	0.003	149
Cabergoline (Dostinex^®^)	13 (8.72%)	7 (7.53%)	6 (10.7%)	0.556	149
Remission (> 30 uG/l)	24 (17%)	9 (10%)	15 (27%)	0.017	142
Follow-up (months) (Median, IQR)	80 [40–154]	89 [39–157]	76 [43–146]	0.812	147

### Cavernous sinus invasion

**Fig. 1:**
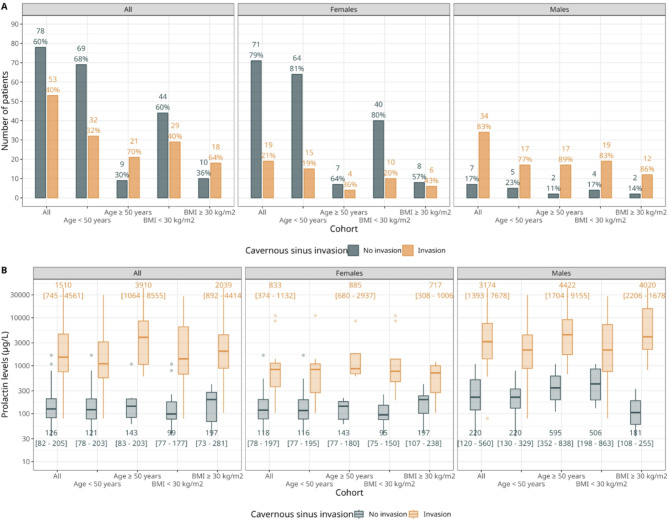
Distribution of the primary outcome (cavernous sinus invasion) stratified according to gender, as well as age- and BMI-related categories. (**A**) Counts and relative frequencies are displayed. (**B**) Distribution of PRL levels on a log-transformed scale, stratified similarly to panel (**A**). Boxplots are shown, along with numerical values of the median and interquartile range.

The subsequent analysis is based on a complete case analysis, wherein only patients with both the outcome (cavernous sinus invasion) and PRL levels are considered (*N* = 131). A detailed distribution of the primary outcome in different subgroups is illustrated in Fig. 1. The incidence of cavernous sinus invasion in female patients closely mirrors the overall distribution in the entire cohort, indicating a predominance of cavernous sinus invasion in male patients, regardless of age and BMI category (Fig. 1A). The corresponding PRL measurements for these subgroups highlight elevated levels in cases of cavernous sinus invasion, with PRL levels in male patients showing large differences of several orders of magnitude (Fig. 1B).

### Prolactin thresholds

**Fig. 2:**
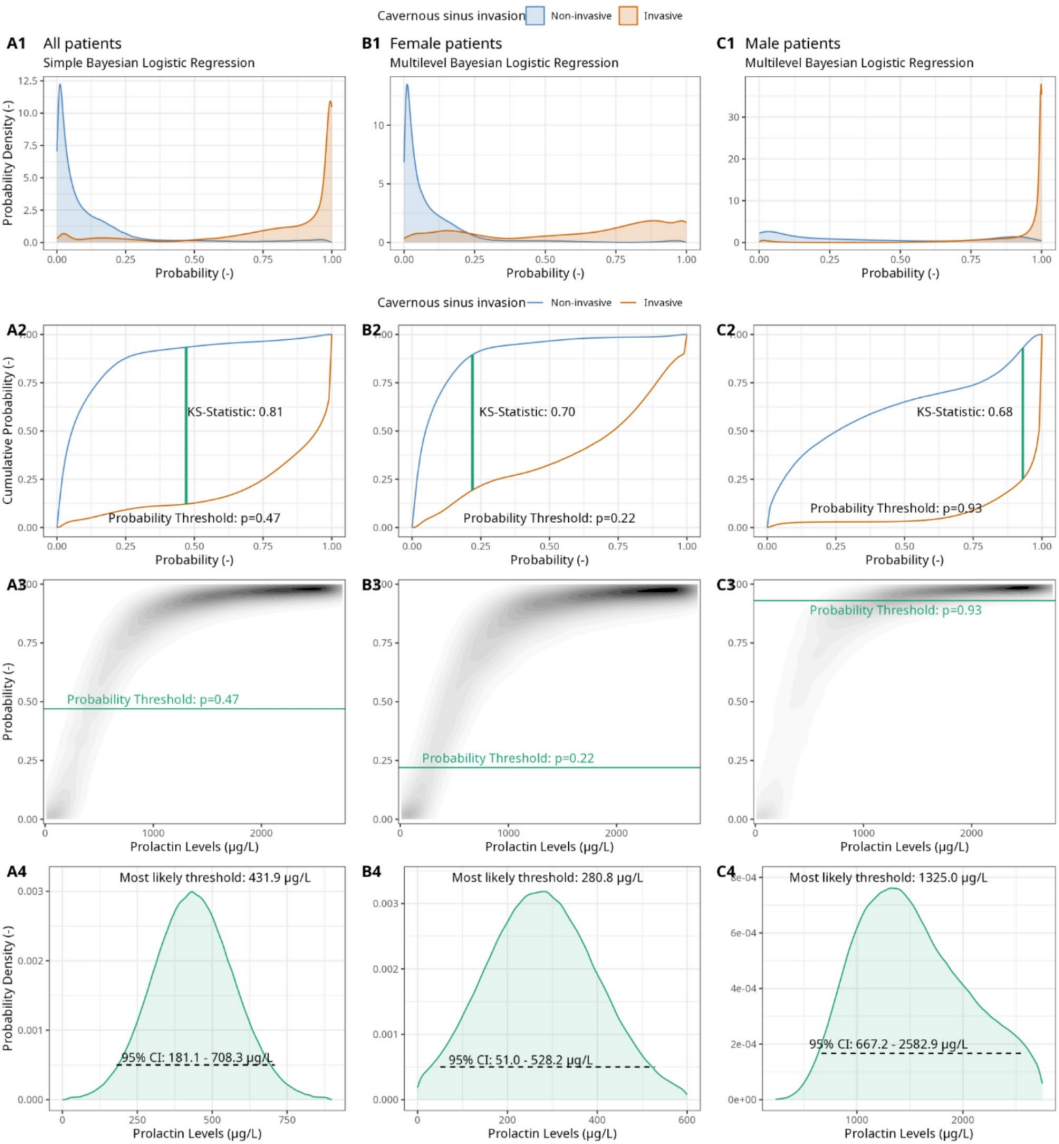
Illustration of the computation of a probabilistic prolactin threshold distribution for adenoma invasion using a simple Bayesian logistic regression framework. (Column A) A Bayesian logistic regression model is fitted to the logarithmic prolactin values of our cohort (*N* = 131), with invasiveness as the outcome. Predicted probability distributions are displayed separately for non-invasive (blue) and invasive (red) adenomas, allowing inspection of the degree of calibration and discrimination between the invasion types (A1). The derivation of an optimal probability threshold based on the cumulative probability distribution of the two invasion classes using the Kolmogorov-Smirnov (K-S) statistic is illustrated (A2). The optimal probability threshold is projected onto a prolactin threshold distribution using the posterior probabilistic distribution of the Bayesian logistic regression (A3). The derived observationally constrained distribution of the optimal prolactin threshold for all patients is illustrated, with the most likely threshold and the 95% credible interval (CI) shown (A4). (Column B) Derivation of a female-specific prolactin threshold. (Column C) Derivation of a male-specific prolactin threshold. A detailed description of the methodology has been provided previously^62^.

The derivation of the PRL threshold is illustrated in Fig. 2 and tabulated in Table 2, both for the entire cohort (column A) and separately for female and male patients (column B for females and column C for males).

**Table 2:** Estimates of prolactin thresholds.

MLE/Median (95%-CI) are shown	Bayesian Regression Framework	Youden Index
All patients		
All patients	431.9 / 437.2 / 181.1–708.3	- / 465.0 (264.0–600.0)
Age < 50 years	404.3 / 404.1 / 146.1–694.3	- / 465.0 (264.0–637.5)
Age ≥ 50 years	454.6 / 450.2 / 136.0–776.3	- / 600.0 (600.0–1704.0)
BMI < 30 kg/m^2^	332.6 / 330.1 / 88.0–592.2	- / 264.0 (264.0–596.2)
BMI ≥ 30 kg/m^2^	571.4 / 672.2 / 241.1–2130.8	- / 600.0 (600.0–1064.0)
Female patients		
All females	280.8 / 276.1 / 51.0–528.2	- / 264.0 (189.8–600.0)
Age < 50 years	367.4 / 377.1 / 111.0–709.3	- / 264.0 (189.8–466.1)
Age ≥ 50 years	264.9 / 293.1 / 41.0–625.2	- / 600.0 (600.0–2374.9)
BMI < 30 kg/m^2^	314.7 / 334.1 / 79.0–680.2	- / 264.0 (189.8–706.0)
BMI ≥ 30 kg/m^2^	314.1 / 319.1 / 57.0–786.3	- / 600.0 (104.1–1064.0)
Male patients		
All males	1325.0 / 1464.5 / 667.2–2582.9	- / 1354.0 (596.2–1718.0)
Age < 50 years	659.7 / 762.3 / 264.1–1739.6	- / 811.6 (596.2–1354.0)
Age ≥ 50 years	2292.2 / 1855.7 / 824.3–2683.0	- / 1510.0 / 666.0–2374.0)
BMI < 30 kg/m^2^	2076.8 / 1904.7 / 824.3–2684.0	- / 1718.0 / 596.2–3130.0)
BMI ≥ 30 kg/m^2^	1250.4 / 1485.5 / 444.2–2632.0	- / 811.6 / 811.6–2374.0)

BMI: Body mass index; CI: Credible interval.

Estimates of PRL thresholds [in µg/L] are derived from the (multilevel) Bayesian logistic regression framework and the Youden Index. The former provides the most likely estimate (MLE), the median estimate, and the 95%-credible interval. The latter offers the median and 95%-confidence interval based on a 1,000-member bootstrap sample.

**Fig. 3:**
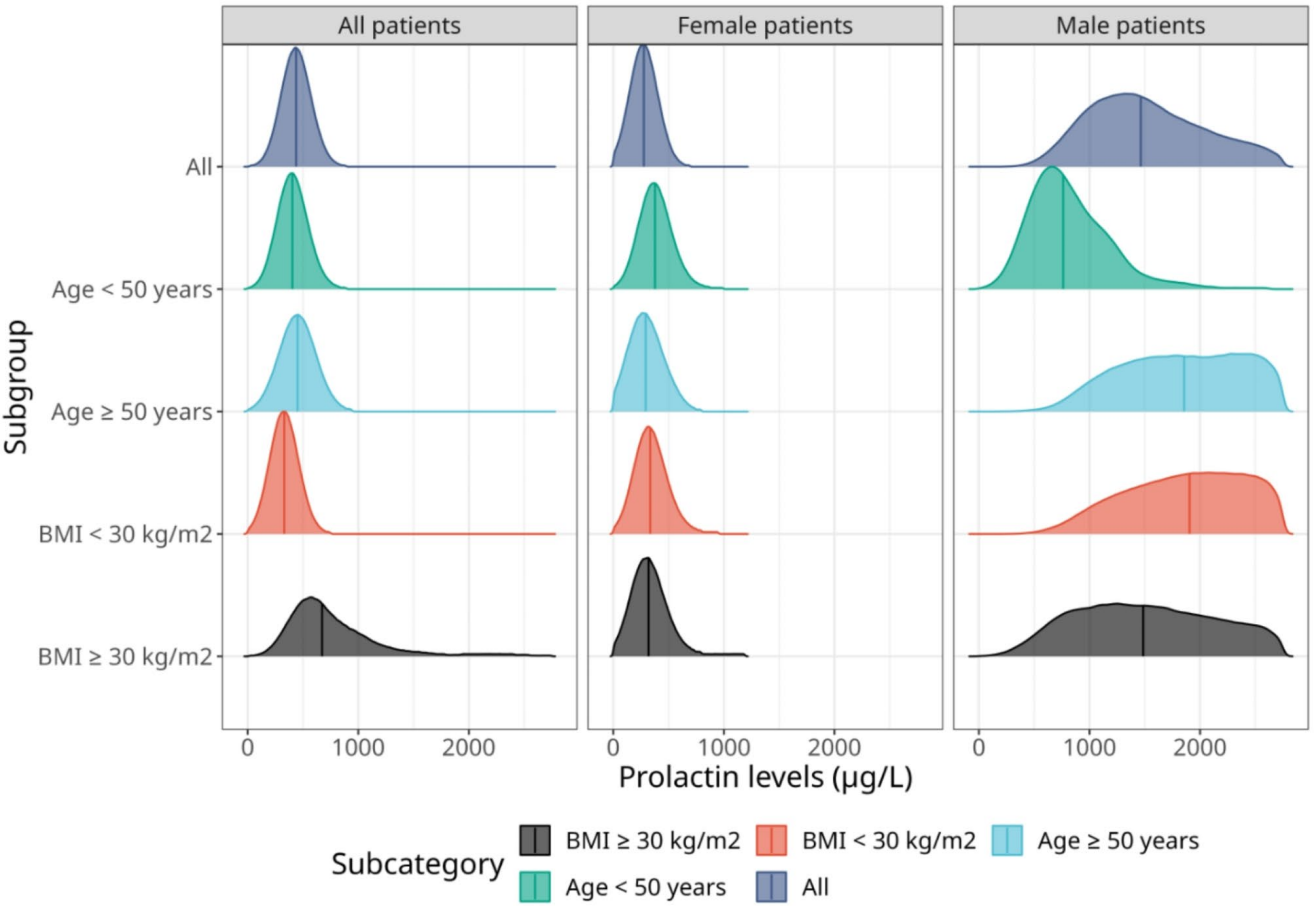
Prolactin threshold distributions for discriminating between non-invasive and invasive adenoma sizes based on a Bayesian logistic regression framework. The solid vertical lines denote the estimated median prolactin threshold.

With respect to the primary outcome, we compute a most likely PRL threshold of 431.9 µg/L (95%-CI: 181.1–708.3 µg/L). Considering gender differences, the methodology yields a threshold of 280.8 µg/L (95%-CI: 51.0–528.2 µg/L) for female patients and 1325.0 µg/L (95%-CI: 667.2–2582.9 µg/L) for male patients, respectively. The smaller sample size of male patients resulted in wider credible intervals compared to the uncertainty related to female-specific thresholds. Figure 3 demonstrates that the threshold estimates for females are similar across age- and BMI-defined subgroups.

In contrast, we observe some subgroup-dependence for male patients: for instance, the threshold for males aged younger than 50 years is lower (367.4 µg/L, 95%-CI: 111.0–709.3 µg/L) than the threshold estimated based on all male patients. Overall, the most likely threshold estimate for the entire cohort and for females and males are similar to the thresholds derived with the Youden Index (Table 2).

### Performance metrics and clinical significance

The prolactin values demonstrate a high discriminatory capacity with respect to cavernous sinus invasion, with AUROC values of 0.95 (95%-CI: 0.90–0.98, entire cohort), 0.93 (95%-CI: 0.85–0.99, female patients), and 0.92 (95%-CI: 0.82–0.99, male patients, Table 3).

**Table 3:** Classification performance.

Subgroup	All patients	Female patients	Male patients
All	0.95 (95%-CI: 0.90–0.98)	0.93 (95%-CI: 0.85–0.99)	0.92 (95%-CI: 0.82–0.99)
Age < 50 years	0.93 (95%-CI: 0.86–0.98)	0.91 (95%-CI: 0.81–0.98)	0.93 (95%-CI: 0.78–0.99)
Age ≥ 50 years	0.97 (95%-CI: 0.89–0.99)	0.99 (95%-CI: 0.99–0.99)	0.93 (95%-CI: 0.80–0.99)
BMI < 30 kg/m^2^	0.95 (95%-CI: 0.89–0.99)	0.98 (95%-CI: 0.93–0.99)	0.80 (95%-CI: 0.53–0.99)
BMI ≥ 30 kg/m^2^	0.93 (95%-CI: 0.81–0.99)	0.79 (95%-CI: 0.47–0.99)	0.80 (95%-CI: 0.56–0.99)

Classification performance, as measured by the area under the operating characteristic curve (AUROC), is stratified according to gender, as well as age and BMI categories. The mean and 95% credible intervals are presented.

**Fig. 4:**
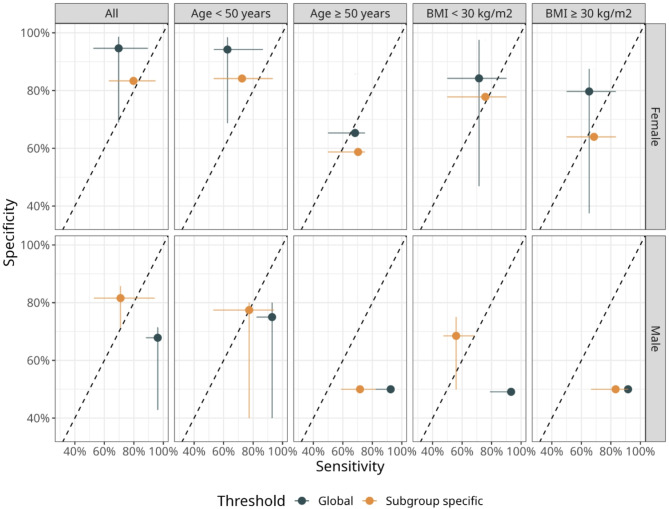
Illustration of the impact of gender-specific classification on sensitivity and specificity. Mean values (points) and 95% credible intervals are displayed.

Moderate AUROC values with large uncertainty of 0.80 (95%-CI: 0.53–0.99) and 0.79 (95%-CI: 0.47–0.99) are found for male patients with BMIs below 30 kg/m^2^ and for female patients with BMIs above 30 kg/m^2^, respectively, which is likely due to the small sample size of those subgroups (Fig. 1A). Figure 4 illustrates the effect of using gender-specific thresholds on sensitivity and specificity estimates for female patients (upper panel) and male patients (lower panel).

The numerical values for all performance metrics are provided in Table 1 of the Supplementary Material. Overall, we find a sensitivity of 0.87 (95%-CI: 0.75–0.96) and a specificity of 0.92 (95%-CI: 0.67–0.96). For females, there is only a marginal effect: for instance, there is a shift towards slightly higher sensitivity values and lower specificity values, from a mean sensitivity and specificity of 0.70 and 0.95 to 0.80 and 0.83, respectively, which likely has only minor clinical impact. However, there is an effect for male patients: when evaluated with a global (i.e., gender-unaware) threshold, there is a high sensitivity of 0.96 (95%-CI: 0.88–0.97) and moderate specificity of 0.68 (95%-CI: 0.43–0.71). In contrast, a sensitivity of 0.71 (95%-CI: 0.53–0.94) and a specificity of 0.82 (95%-CI: 0.71–0.86) are observed. Overall, Fig. 4 highlights that the gender-specific thresholds result in more balanced sensitivity-specificity performances compared to thresholds derived from the entire cohort.

## Discussion

Our study unveils a notable disparity in PRL thresholds between genders: (i) females exhibit lower thresholds for adenoma invasiveness compared to males, demonstrating consistent performance across various age and obesity categories. (ii) In contrast, male-specific thresholds show sensitivity to age and obesity, indicating lower thresholds in younger, obese males.

Dopamine agonists (DAs) remain the first-line treatment for prolactinomas, particularly in patients with macroadenomas Knosp grade ≥ 1 ^21^ or those planning pregnancy^22^. While bromocriptine has an established safety profile in preconception^23^, data on cabergoline remain more limited but have not indicated increased risks^23^. In macroprolactinomas, treatment—whether medical or surgical—is often necessary before pregnancy to reduce tumor size and mitigate potential tumor growth during gestation^24^. Thereby, an individualized approach is crucial, particularly for tumors abutting the optic chiasm, where continued DA therapy may be warranted^22^. However, surgical data in females of reproductive age remain limited. A study of women under 40 years of age undergoing transsphenoidal surgery reported postoperative normoprolactinemia in many cases, with better outcomes in non-invasive adenomas^25^. Hypopituitarism was observed in a small percentage of them, highlighting the need to weigh the potential risk of surgical hypogonadotropic hypogonadism against the benefits of surgery^25^. Although morbidity, mortality and rates of new endocrinopathies following upfront surgery are limited^26^, they are not nonexistent. In contrast, DAs can be easily administered and monitored and are usually well tolerated^27^. Given that long-term DA therapy is associated with impulse control disorders (ICDs)^28,29^, particularly hypersexuality in males^30^, the potential for mental health destabilization in patients dependent on DAs must also be considered. Careful monitoring is especially warranted in high-risk individuals, such as those with a history of gambling, smoking, or alcohol use^30^. Even though these side effects are rare, they can still lead to significant psychosocial consequences^31,32^, which patients often delay reporting due to experiencing profound shame.

The trend towards offering first-line surgery to younger patients with microprolactinomas or non-cavernous sinus-invading macroprolactinomas aligns with the Pituitary Society’s 2024 consensus statement^17^. They further strengthened diagnostic algorithms by identifying PRL levels > 200 ng/ml as indicative of prolactinoma rather than other diagnoses in the evaluation of hyperprolactinemia^17^. This emphasizes the importance of establishing straightforward PRL thresholds in clinical practice to define invasiveness and inform treatment decisions between TSS and DA therapy for long-term cure without the need for continuous DAs. Namely, elevated baseline PRL levels and cavernous sinus invasion are consistent factors associated with prolactinoma diagnosis and treatment outcomes^33^. A lower PRL threshold for upfront surgery was particularly considered in individuals deemed likely to benefit from TSS instead of persistent DA therapy in the long term^34^. This was mostly attributed to microprolactinomas or adenomas not infiltrating the cavernous sinus^1,14,18^.

The need for gender-specific thresholds in particular arises from inherent differences in PRL levels between males and females, compounded by the varying impacts of age and obesity. Namely, global thresholds fail to capture these nuances, necessitating tailored thresholds to better guide treatment decisions. In essence, gender-specific thresholds may offer a more refined approach to improve treatment selection and the likelihood of achieving long-term remission. Our findings demonstrate significant gender differences in PRL thresholds for adenoma invasiveness, highlighting their potential role in shared decision-making for first-line therapy. PRL levels show a strong discriminatory capacity for differentiating cavernous sinus invasion, with mean AUROC values ranging from 0.92 to 0.95. Importantly, evidence suggests gender-specific thresholds of 1325 µg/L for men and 281 µg/L for women, respectively. Gender-specific differences in prolactinomas are well-documented phenomena^22,35^. In men, macroprolactinomas are frequently diagnosed at an older age than in women, who mostly present with microprolactinomas^19,36^. Given the obvious clinical features of amenorrhea or galactorrhea in women, the prolonged period of often oligosymptomatic men, such as lower libido, hinders early diagnosis^8,9,15,37^. This corroborates that gender is an important determinant of tumor size and subsequent invasion^14^. Additionally, prolonged hyperprolactinemia and delayed diagnosis contribute to older age at diagnosis in men^37^. Namely, macroprolactinomas exhibit prolonged time of hyperprolactinemia- and thus older age at diagnosis^8,38^, as the lower rates of baseline PRL levels in women probably reflects on the shorter disease duration in more evident symptoms in women^37,39^. In addition, it has been shown that serum PRL rose slightly with increasing age in men^40^. In this context, this study goes a step further and unveils a notable disparity in PRL thresholds between genders, with females exhibiting lower thresholds for adenoma invasiveness compared to males. Moreover, these female-specific thresholds demonstrate consistent performance across various age and obesity categories. Conversely, male-specific thresholds exhibit sensitivity to age and obesity, indicating lower thresholds in younger (< 50 years), obese (BMI > 30 kg/m^2^) males. This is an intriguing finding. In general, obesity in patients with hyperprolactinemia is not an uncommon phenomenon, as high PRL levels can contribute to obesity^20,41^, although the exact mechanism remains unclear^42,43^. The lower thresholds in obese vs. non-obese men might account for the fact that longer exposure to hyperprolactinemia is related to higher BMI levels in patients with macroprolactinomas with prompted diagnosis work-up^44^. As there is a positive correlation between tumor volume and serum PRL levels^2^, the higher thresholds are gender specific and dependent- in men- on age and obesity. This must be taken into consideration when guiding individuals toward an optimal therapy in clinical practice.

With respect to clinical impact of the gender-specific PRL thresholds compared to cohort-wide thresholds, we find that the former results in more balanced specificity-sensitivity values. When evaluated in female patients only, a cohort-wide PRL threshold results in a high specificity of 0.95, suggesting the possibility to rule in cavernous sinus invasion in case of a positive test. In contrast, a cohort-wide PRL threshold results in a high sensitivity of 0.96, suggesting the possibility to rule out cavernous sinus invasion in case of a negative test result. Thus, the more balanced gender-specific thresholds provide additional benefit for clinical decision making for scenarios with these two possible outcomes.

In summary, our findings underscore the importance of personalized PRL threshold values, which not only offer additional diagnostic information but also present a more balanced sensitivity-specificity profile. By facilitating the identification of prolactinoma patients who stand to benefit most from upfront surgery, these personalized thresholds hold promise for achieving long-term remission and reducing reliance on dopamine agonists as part of the treatment regimen.

### Study limitations

The study is subject to several inherent limitations that warrant consideration. Firstly, its single-center design may limit the generalizability of the findings to broader populations. Additionally, the specific gender distribution within the study cohort may introduce bias and restrict the applicability of the results to other demographic groups. Furthermore, while the database is prospectively maintained, the study design is retrospective. PRL levels can be influenced by various factors; higher PRL levels may not only indicate cavernous sinus invasion but also simply reflect adenoma size, adding complexity to the interpretation of results. In addition, a small number of tumors measuring exactly 10 mm may have been misclassified as microadenomas, as we considered tumors in the 1–10 mm range as microadenomas, which is not in line with the 2023 Pituitary Society consensus statement that defines macroadenomas as including tumors exactly 10 mm^17^. Nonetheless, our research boasts a significant advantage due to the exceptional uniformity of our dataset, spanning a quarter-century, featuring consistent indications, treatment methods, and monitoring procedures. Another limitation in this regard is the potential impact of evolving MRI technology on the assessment of invasion. Since the first patient underwent surgery in 1996, there have been significant advancements in MRI techniques, including the introduction of dynamic sella MRI, which was not available for all patients throughout the study period. Yet, the limitations underscore the need for cautious interpretation and highlight avenues for future research to validate and expand upon the current findings.

## Conclusions

For the first time, we demonstrate that female-specific PRL thresholds for identifying adenoma invasiveness are lower compared to male-specific thresholds and show no significant dependence on age and obesity. In contrast, male-specific thresholds are sensitive to age and obesity, with lower thresholds observed in young, obese men. Our findings suggest that personalized PRL threshold values are associated with adenoma invasiveness and offer additional information, providing a more balanced sensitivity-specificity profile. They may add value by assisting in the identification of prolactinoma patients, both men and women, who could benefit from upfront surgery, potentially leading to long-term remission and reduced dependence on DAs.

## Methods

### Study design

The data was gathered from a comprehensive series sourced from a prospectively maintained database of patients diagnosed with prolactinoma between January 1996 and November 2017. All patients fulfilled the diagnostic criteria for a PRL-secreting pituitary adenoma. These included the immunoradiometric PRL assay with serum dilution in order to overcome the high-dose PRL hook effect^45–48^. The established cutoff prolactin level for distinguishing between prolactinoma and nonfunctioning adenomas with a stalk effect or dual secreting GH-PRL adenomas is 200 µg/L, indicative of prolactinoma^4,17,22^.

Partial hypopituitarism was defined as deficient secretion of one or more pituitary hormones. Secondary adrenal insufficiency was diagnosed based on low serum cortisol levels (< 50 nmol/L) or normal cortisol levels with inadequate responses to the adrenocorticotropin (ACTH) stimulation test or insulin tolerance test^49–51^. Secondary hypothyroidism was identified by low-normal thyroid-stimulating hormone (TSH) levels and low free thyroxin (FT4) levels. A deficiency in gonadotropin or central hypogonadism was confirmed if there were low-normal levels of gonadotropins alongside low total testosterone levels. Standard body mass index (BMI) calculations were carried out for all patients^52^, with BMI values falling between 19 and 25 kg/m² considered within the normal range.

### Radiological assessment

A dynamic MRI of the sellar region was performed using a combination of proton density and T2-weighted scans of the entire brain. This included both unenhanced and contrast-enhanced scans, with overlapping slices in both sagittal and coronal planes^49–51^. Microadenomas were identified by diameters ranging from 1 to 10 mm, while macroadenomas were ≥ 10 mm.^17^ The Knosp classification system was utilized to determine adenoma infiltration into the cavernous sinus, with crossing the median carotid line indicating infiltration (i.e., Knosp grade ≥ 1)^53,54^. The invasion grading was independently assessed by a neuroradiologist during the diagnostic workup. As for this study, data were extracted from written reports and dichotomized based on the presence or absence of noted invasion.

### Treatment strategy

For all patients, the decision to proceed with surgery was made during an interdisciplinary pituitary tumor board meeting, aiming to avoid long-term dependence on DA therapy. This decision was also discussed with the patient, considering their preference for surgical treatment over prolonged DA therapy^17,55^. The decision regarding the primary treatment strategy was independent of financial considerations, as both medical and surgical treatments are covered for all residents by health insurance in Switzerland. Prior DA therapy was a contraindication for upfront surgery. TSS was conducted using a transseptal, transsphenoidal microsurgical approach, with sellar reconstruction^56^. DAs were gradually tapered off 24 months after initiating medical therapy once PRL levels were normalized^57,58^. Recurrence was defined as an elevation in PRL levels above the normal range (> 20 µg/L) during the last follow-up period after a previous remission, regardless of radiological findings^59–61^.

### Statistical analysis

#### Descriptive statistics

Categorical variables are summarized by counts and frequencies, while numerical variables are described with median and interquartile range. For exploratory purposes only, univariable (unadjusted) group comparisons using standard statistical tests (chi-square test for categorical variables and unpaired two-samples Wilcoxon test for numerical variables) of patient characteristics with respect to the primary outcome are computed and presented in Table 1.

The dataset is indicated in Table 1. No imputation was performed, and the results are based on a complete-case analysis.

### Computation of prolactin thresholds

Thresholds for prolactin values concerning cavernous sinus invasion were determined using a Bayesian mixed-effect logistic regression model. The model incorporates log-transformed (base 10) serum prolactin as a fixed effect, a random offset for gender, and cavernous sinus invasion as a binary outcome. By combining the posterior distributions of the model parameters with the Kolmogorov–Smirnov (KS) statistic, prolactin thresholds can be derived for the entire cohort and specifically for female and male patients. This method was recently introduced to derive prolactin thresholds concerning prolactinoma size, where a detailed method description, including specifics regarding the derivation of the posterior distribution with Markov chain Monte Carlo (MCMC) samples, is provided^62^. Regarding statistical inference, we denote the uncertainty of the observationally constrained quantities of interest with 95% credible intervals (CI). In addition to the thresholds derived with the Bayesian mixed-effect logistic regression model, optimal thresholds derived with the Youden Index and their 95% confidence intervals, are also provided for comparison.

The Bayesian mixed-effect logistic regression model was employed to compute subgroup-specific prolactin thresholds, namely concerning age (below or above 50 years) and obesity (below or above 30 kg/m²).

### Threshold evaluation

The clinical utility of the gender-specific PRL threshold was assessed by comparing traditional performance metrics (sensitivity, specificity, positive predictive value, negative predictive value) evaluated for a specific subgroup – for instance, female patients - using either a global threshold (i.e., a PRL threshold unaware of potential gender differences) or a subgroup-specific threshold. Furthermore, the area under the receiver operating characteristic curve (AUROC) was calculated to measure the discriminatory capacity of PRL levels regarding cavernous sinus invasion, both for the entire cohort and for the aforementioned subgroups, such as males with a body mass index below 30 kg/m².

### Statistical software

All computations were conducted using R version 4.2.3^63^. The Bayesian mixed-effect logistic regression model was implemented with the R-package *rstan*^64^.

## Electronic supplementary material

Below is the link to the electronic supplementary material.

Supplementary Material 1

## Data Availability

The dataset is available upon request. Please contact (lukas.andereggen@unibe.ch).

## Declarations

### Word and figures count

3089 words in manuscript, 4 figure, 3 tables.
