# Supplementary material for: Gender differences in prolactin thresholds and their association with lactotroph adenoma invasiveness for potential treatment considerations
Source: Sci Rep. 2025 Mar 20;15:9598. doi: 10.1038/s41598-025-90250-6 (PMC11926263; doi:10.1038/s41598-025-90250-6)
Supplement: Supplementary file 1 — Supplementary Material 1 [file 41598_2025_90250_MOESM1_ESM.docx]

**Supplementary Material**

| **Cohort** | **Subgroup** | **Threshold**  **Type** | **Sensitivity** | **Specificity** | **Positive Predictive Value** | **Negative Predictive Value** |  |
| --- | --- | --- | --- | --- | --- | --- | --- |
| All | All | Global | 0.87  (95%-CI: 0.75 - 0.96) | 0.92  (95%-CI: 0.67 - 0.96) | 0.89  (95%-CI: 0.66 - 0.94) | 0.91  (95%-CI: 0.85 - 0.95) |  |
| Female | All | Global | 0.70  (95%-CI: 0.53 - 0.89) | 0.95  (95%-CI: 0.69 - 0.99) | 0.82  (95%-CI: 0.45 - 0.92) | 0.92  (95%-CI: 0.89 - 0.97) |  |
| Male | All | Global | 0.96  (95%-CI: 0.88 - 0.97) | 0.68  (95%-CI: 0.43 - 0.71) | 0.94  (95%-CI: 0.89 - 0.94) | 0.80  (95%-CI: 0.56 - 0.83) |  |
| Female | All | Subgroup specific | 0.80  (95%-CI: 0.63 - 0.95) | 0.83  (95%-CI: 0.27 - 0.97) | 0.66  (95%-CI: 0.24 - 0.88) | 0.94  (95%-CI: 0.91 - 0.98) |  |
| Male | All | Subgroup specific | 0.71  (95%-CI: 0.53 - 0.94) | 0.82  (95%-CI: 0.71 - 0.86) | 0.96  (95%-CI: 0.94 - 0.96) | 0.42  (95%-CI: 0.30 - 0.71) |  |
| *Age categories* | | | | | | | |
| Female | Age < 50 years | Global | 0.63  (95%-CI: 0.53 - 0.87) | 0.94  (95%-CI: 0.69 - 0.98) | 0.77  (95%-CI: 0.41 - 0.89) | 0.92  (95%-CI: 0.90 - 0.96) |  |
| Female | Age < 50 years | Subgroup specific | 0.72  (95%-CI: 0.53 - 0.93) | 0.84  (95%-CI: 0.27 - 0.98) | 0.62  (95%-CI: 0.21 - 0.89) | 0.93  (95%-CI: 0.90 - 0.98) |  |
| Female | Age ≥ 50 years | Global | 0.68  (95%-CI: 0.50 - 0.75) | 0.65  (95%-CI: 0.29 - 0.86) | 0.64  (95%-CI: 0.45 - 0.80) | 0.85  (95%-CI: 0.78 - 0.88) |  |
| Female | Age ≥ 50 years | Subgroup specific | 0.70  (95%-CI: 0.50 - 0.75) | 0.59  (95%-CI: 0.14 - 0.86) | 0.57  (95%-CI: 0.36 - 0.80) | 0.86  (95%-CI: 0.78 - 0.88) |  |
| Male | Age < 50 years | Global | 0.93  (95%-CI: 0.82 - 0.94) | 0.75  (95%-CI: 0.40 - 0.80) | 0.93  (95%-CI: 0.84 - 0.94) | 0.76  (95%-CI: 0.57 - 0.80) |  |
| Male | Age < 50 years | Subgroup specific | 0.77  (95%-CI: 0.53 - 0.94) | 0.77  (95%-CI: 0.40 - 0.80) | 0.93  (95%-CI: 0.84 - 0.94) | 0.57  (95%-CI: 0.38 - 0.80) |  |
| Male | Age ≥ 50 years | Global | 0.92  (95%-CI: 0.82 - 0.94) | 0.50  (95%-CI: 0.50 - 0.50) | 0.94  (95%-CI: 0.94 - 0.94) | 0.46  (95%-CI: 0.25 - 0.50) |  |
| Male | Age ≥ 50 years | Subgroup specific | 0.72  (95%-CI: 0.59 - 0.82) | 0.50  (95%-CI: 0.50 - 0.50) | 0.93  (95%-CI: 0.93 - 0.94) | 0.30  (95%-CI: 0.22 - 0.40) |  |
| *BMI categories* | | | | | | | |
| Female | BMI < 30 kg/m2 | Global | 0.71  (95%-CI: 0.50 - 0.90) | 0.84  (95%-CI: 0.47 - 0.98) | 0.66  (95%-CI: 0.32 - 0.90) | 0.93  (95%-CI: 0.89 - 0.97) |  |
| Female | BMI < 30 kg/m2 | Subgroup specific | 0.76  (95%-CI: 0.50 - 0.90) | 0.78  (95%-CI: 0.11 - 0.98) | 0.59  (95%-CI: 0.20 - 0.90) | 0.94  (95%-CI: 0.89 - 0.98) |  |
| Female | BMI ≥ 30 kg/m2 | Global | 0.65  (95%-CI: 0.50 - 0.83) | 0.80  (95%-CI: 0.38 - 0.88) | 0.74  (95%-CI: 0.50 - 0.80) | 0.78  (95%-CI: 0.73 - 0.80) |  |
| Female | BMI ≥ 30 kg/m2 | Subgroup specific | 0.69  (95%-CI: 0.50 - 0.83) | 0.64  (95%-CI: 0.25 - 0.88) | 0.64  (95%-CI: 0.43 - 0.80) | 0.77  (95%-CI: 0.67 - 0.80) |  |
| Male | BMI < 30 kg/m2 | Global | 0.93  (95%-CI: 0.79 - 0.95) | 0.49  (95%-CI: 0.25 - 0.50) | 0.90  (95%-CI: 0.86 - 0.90) | 0.63  (95%-CI: 0.33 - 0.67) |  |
| Male | BMI < 30 kg/m2 | Subgroup specific | 0.56  (95%-CI: 0.47 - 0.68) | 0.68  (95%-CI: 0.50 - 0.75) | 0.91  (95%-CI: 0.87 - 0.93) | 0.32  (95%-CI: 0.29 - 0.36) |  |
| Male | BMI ≥ 30 kg/m2 | Global | 0.92  (95%-CI: 0.92 - 0.92) | 0.50  (95%-CI: 0.50 - 0.50) | 0.92  (95%-CI: 0.92 - 0.92) | 0.67  (95%-CI: 0.67 - 0.67) |  |
| Male | BMI ≥ 30 kg/m2 | Subgroup specific | 0.83  (95%-CI: 0.67 - 0.92) | 0.50  (95%-CI: 0.50 - 0.50) | 0.92  (95%-CI: 0.92 - 0.92) | 0.53  (95%-CI: 0.33 - 0.67) |  |

**Supplementary Table SM1**. Performance metrics for estimating the binary outcome cavernous sinus invasion with observationally derived prolactin thresholds from a Bayesian mixed-effect logistic regression model (see Methods).
